# Sphingolipid lysosomal storage diseases: from bench to bedside

**DOI:** 10.1186/s12944-021-01466-0

**Published:** 2021-05-03

**Authors:** Muna Abed Rabbo, Yara Khodour, Laurie S. Kaguni, Johnny Stiban

**Affiliations:** 1 Department of Biology and Biochemistry, Birzeit University, P.O. Box 14, Ramallah, West Bank 627 Palestine; 2 Department of Biochemistry and Molecular Biology, Michigan State University, East Lansing, MI USA

**Keywords:** sphingolipids, lysosomal storage diseases, inborn errors of metabolism, neurological diseases, sphingolipidoses, Gaucher, Krabbe, gangliosidosis, Fabry

## Abstract

Johann Ludwig Wilhelm Thudicum described sphingolipids (SLs) in the late nineteenth century, but it was only in the past fifty years that SL research surged in importance and applicability. Currently, sphingolipids and their metabolism are hotly debated topics in various biochemical fields. Similar to other macromolecular reactions, SL metabolism has important implications in health and disease in most cells. A plethora of SL-related genetic ailments has been described. Defects in SL catabolism can cause the accumulation of SLs, leading to many types of lysosomal storage diseases (LSDs) collectively called sphingolipidoses. These diseases mainly impact the neuronal and immune systems, but other systems can be affected as well. This review aims to present a comprehensive, up-to-date picture of the rapidly growing field of sphingolipid LSDs, their etiology, pathology, and potential therapeutic strategies. We first describe LSDs biochemically and briefly discuss their catabolism, followed by general aspects of the major diseases such as Gaucher, Krabbe, Fabry, and Farber among others. We conclude with an overview of the available and potential future therapies for many of the diseases. We strive to present the most important and recent findings from basic research and clinical applications, and to provide a valuable source for understanding these disorders.

## Introduction

As essential components of membranes that play vital roles in a variety of signaling cascades, sphingolipids (SLs) represent a hot topic of metabolic research [1]. SLs not only have structural functions but also play other vital roles in cellular homeostasis, adhesion, signaling, senescence, development, and death [2, 3]. SLs are also involved in the pathology of several immune and neurological diseases [4].

**Fig. 1:**
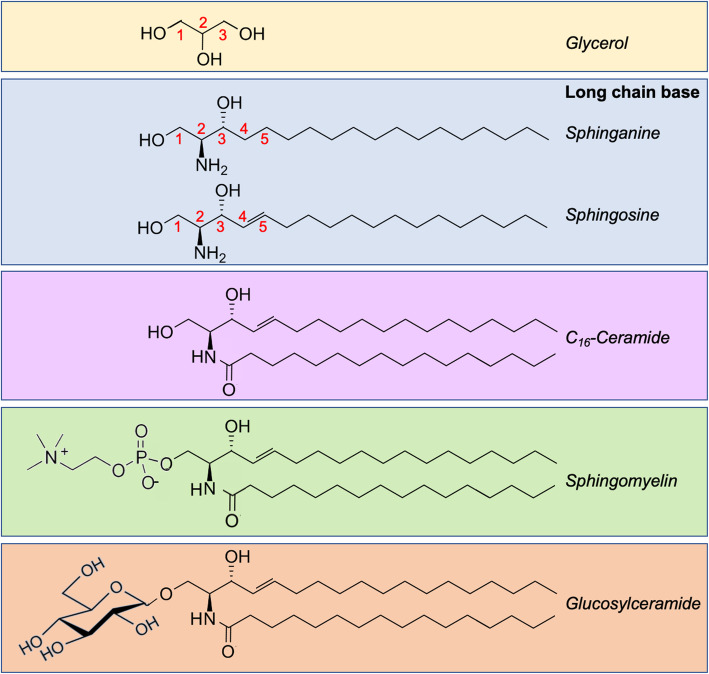
Common structures of representative SLs. SLs contain a long-chain base whether sphinganine (saturated) or sphingosine (monounsaturated at C4). *N-*acylation at the amino group on C2 creates ceramide, which may consist of varying number of carbon atoms. The representative ceramide shown here is palmitoyl-ceramide that contains 16 carbons. The addition of a phosphocholine at C1 creates the parent phosphosphingolipid sphingomyelin, and glycosylation at the same carbon generates glucosylceramide

SLs are a major class of lipids that differ from glycerolipids in having a long-chain base backbone (sphinganine or sphingosine, Sph) in lieu of glycerol (Fig. 1). An amide linkage joins a fatty acyl group to the amino nitrogen of the long-chain base, forming the second leg of the hydrophobic tail in the molecule, creating ceramide (Cer). Cer is the parent SL that can serve as the metabolic hub for the generation of other SLs [3].

Cells generate many Cer species differing in their chain length, ranging from 14 to 32 carbons in mammals [5]. This contributes to the first layer of heterogeneity among SLs. Another layer of variation arises from different attached head groups [6]. Depending on the head groups, sphingolipids can be classified into phosphosphingolipids (*e.g*., sphingomyelin (SM)) and glycosphingolipids (GSLs). SMs are highly abundant in the myelin sheath surrounding the axonal regions of neural cells [7]. GSLs, on the other hand, are more structurally diverse, and contain one or more sugars attached to the Cer moiety. In mammalian cells, the most commonly attached sugars are glucose, galactose, *N*-acetylglucosamine, *N*-acetylgalactosamine, sialic acid, and fucose [2, 8]. GSLs can be categorized into four subtypes: cerebrosides, sulfatides, globosides, and gangliosides. Cerebrosides have a single sugar attached to the Cer [7]. Sulfatides have an additional sulfate attached to the cerebroside [7]. Sulfatides are thought to participate in myelin formation and maintenance, in addition to neural cell differentiation [8]. Globosides and gangliosides contain a more complex oligosaccharide attached to the Cer moiety; gangliosides  have a negatively-charged sialic acid residue on their head group [7], whereas globosides lack this residue and hence are neutral at pH 7 [8].

**Fig. 2:**
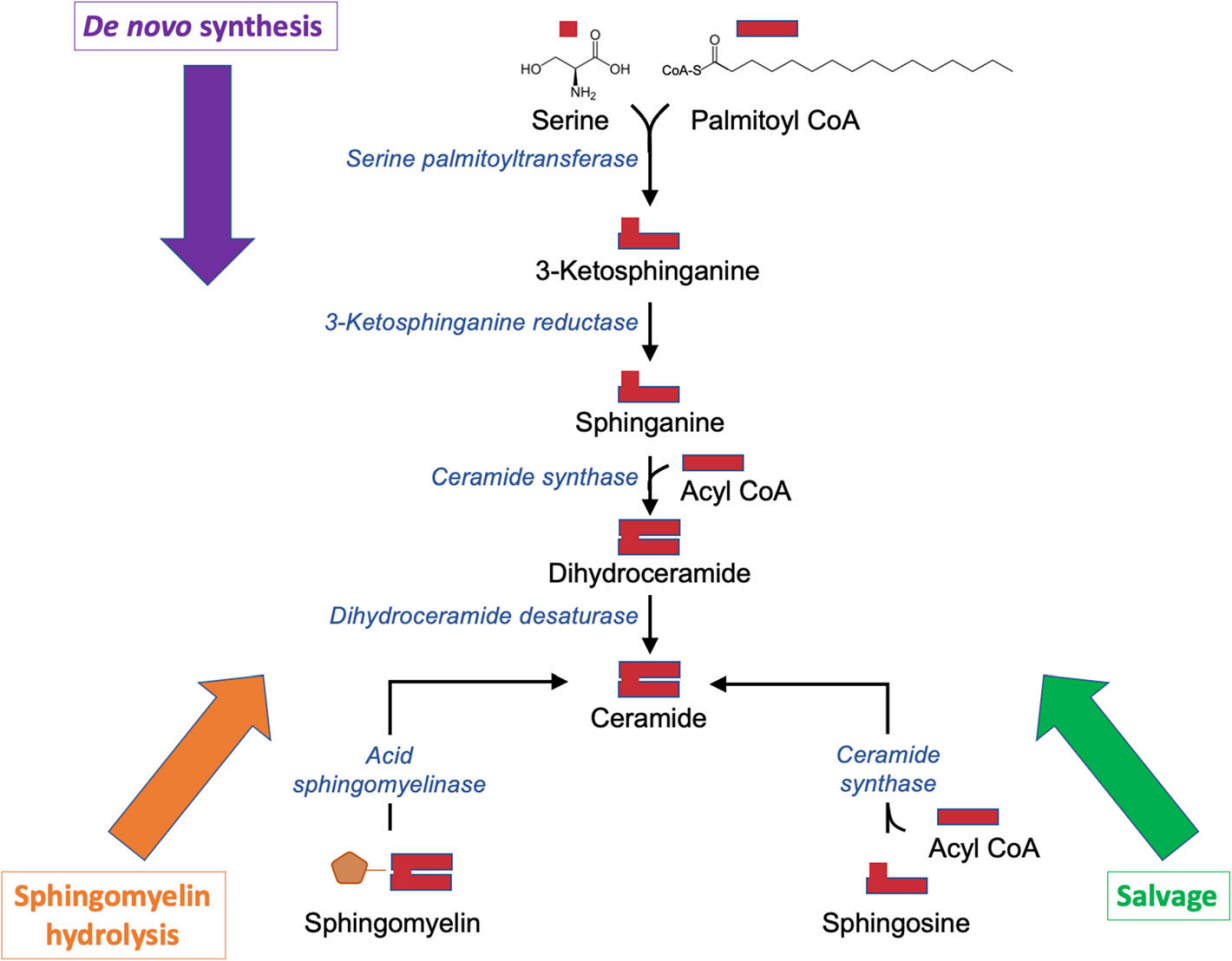
Three general pathways for the generation of Cer. In mammalian cells, Cer is biosynthesized *de novo* or generated by catabolism of complex SLs. In the *de novo* synthesis pathway (purple block arrow), a four-enzyme sequence culminates in the formation of Cer from the amino acid serine and palmitoyl-CoA. This pathway is located in the ER. Sphingomyelin can be hydrolyzed to Cer in the SM hydrolysis pathway (orange block arrow), which is a one-enzyme step. (Degradation of other complex SLs is not shown.) Alternatively, Cer can be produced in the salvage pathway (green block arrows), through the acylation of Sph by the ceramide synthase family of enzymes. The red blocks represent cartoons of the possible structures of the molecules. It should be noted that the acyl-chain length of Cer can vary greatly

Irrespective of the type, SL biosynthesis occurs via the same pathways. SLs can be produced *de novo* from the condensation of serine and palmitoyl-CoA in the endoplasmic reticulum (ER), through a series of reactions culminating in the generation of Cer (Fig. 2), which can have several fates [3]. Alternatively, the salvage pathway regenerates Cer from Sph and fatty acyl-CoAs. Lysosomal degradation of GSLs is required for the re-utilization of their products in salvage pathways [9]. A number of human genetic disorders of SL biosynthesis have been described [10]. One of the best-documented examples is the adult-onset, hereditary sensory and autonomic neuropathy that is caused by a defect in the first enzyme of SL biosynthesis, serine palmitoyltransferase [11].

Catabolism of complex SLs is also a source of Cer generation. Most complex membrane lipids are catabolized through the endosomal/ lysosomal membrane digestion system, where the degradation products are re-utilized in salvage pathways, achieving eventual membrane homeostasis. Defects in the proteins and enzymes needed for lysosomal degradation can lead to a wide range of inherited lysosomal storage disorders, LSDs. In LSDs, the lysosome cannot degrade a specific molecule, leading to its accumulation along with other related molecules [12]. LSDs are categorized into five main families: mucolipidoses, mucopolysaccharidoses, sphingolipidoses, glycoprotein, and glycogen storage diseases, depending on the type of the primary stored compound [1].

Sphingolipidoses comprise a whole group of diseases caused by defects in the sequential lysosomal SL degradation pathway [13]. In general, sphingolipidoses have an incidence of approximately 1 in 10,000 individuals. Although this represents a low incidence in most populations, certain populations, especially those that are relatively isolated either geographically or culturally, have a substantially higher incidence [14, 15]. Such disorders cause critical membrane impairment, and hence affect the survival and growth of most cells, especially neural cells. As a result, neurodegeneration, along with other visceral complications [16], are significant characteristics of many sphingolipidoses [17]. Sphingolipidoses have a multitude of neurological and immunological manifestations, and these diseases have been studied widely as new therapeutic approaches have become available [18].

Sphingolipidoses have many clinical manifestations in a variety of organ systems. The cardiovascular system, for instance, is also affected by some of these diseases. GM1 gangliosidosis exhibits cardiovascular lesions including cardiomegaly and diffuse, nodular thickening of the mitral and tricuspid valves [19], while Sandhoff disease patients experience cardiomegaly and mitral regurgitation [20]. Additionally, a subtype of Gaucher disease is defined by cardiac involvement with aortic and valvular calcification [21]. Fabry disease shows severe effects in the health of the cardiovascular system. Cardiovascular manifestations begin with mitral insufficiency in the pediatric period, followed by left ventricular hypertrophy, congestive heart failure, anginal pain, hypertension, and myocardial infarction in adolescence and adulthood caused by progressive globotriaosylceramide (Gb3) accumulation in the myocardial cells, coronary arteries, the valvular tissue, and the atrioventricular conduction system [20, 21].

## Overview of SL Catabolism

Despite being structurally and functionally diverse, SL biosynthesis and catabolism are both governed by a network of interconnected pathways diverging from a single common starting point, and converging into a common catabolic pathway [22]. Cer serves as a metabolic hub, as it occupies the center of both synthetic and catabolic pathways [23]. SL homeostasis in the cell is tightly regulated through multiple pathways [24]. These pathways may have compensatory functions in some cases in which defective enzymes result in multiple responses to SL imbalance. Consequently, an understanding of SL metabolic networks contributes to greater understanding of the LSDs and subsequent therapeutic design [24, 25]. Because lipids cannot be excreted as readily as hydrophilic molecules, the absence of any single enzyme functioning in the coordinated breakdown pathways of complex SLs leads to the accumulation of lipids inside the cell. In fact, defects in these catabolizing enzymes, especially lysosomal hydrolases, are responsible for a considerable number of LSDs [22].

Membrane GSLs can reach endosomal/ lysosomal compartments via autophagy, endocytosis, or phagocytosis [26, 27]. Inside the lysosome, luminal vesicles are formed by successive budding and fission steps, and the lipid composition of these vesicles is controlled by an endosomal lipid-sorting complex [28]. Membrane-stabilizing sterols, including cholesterol (Chol), are sorted by two sterol-binding proteins, NPC1, and NPC2 [1]. GSLs are then degraded sequentially on the surface of intra-lysosomal vesicles. Lysosomal hydrolases are responsible for attacking specific bonds, and cleave single monosaccharide molecules from the non-reducing ends in a stepwise manner [1]. However, the soluble hydrolases cannot attack gangliosides and GSLs directly due to their hydrophobic nature. Thus, their degradation needs more complex cooperation between the hydrolases and other membrane-perturbing, and lipid-binding proteins, as well as glycoprotein cofactors and SL activator proteins [1, 27]. SL activator proteins are encoded by two genes: GM2-activator protein (GM2A) and prosaposin, a precursor that produces saposins (Saps) A-D upon post-translational modifications [29–34]. In addition, the polycationic nature of soluble hydrolases requires the anionic environment of the intra-lysosomal membranes at pH 5, which is provided by bis(monoacylglycero)phosphate (BMP), dolichol-phosphate, and phosphatidylinositol. Together, they attract soluble hydrolases to the GSL-containing membranes to facilitate degradation [1, 9].

SM degradation occurs via the action of the sphingomyelinase (SMase) family of enzymes that catalyze the hydrolysis of the phosphocholine head group [22]. SMases fall into three main categories: alkaline SMases that are expressed exclusively in the intestines and liver, and work on dietary SM [35]. Neutral SMases, whose functions are not fully understood, may play roles in inflammatory signals, cell growth, and survival [36]. Acid SMases (aSMase) predominantly metabolize SM present in intra-lysosomal membranes. aSMases can also be excreted to catabolize SM-containing lipoproteins found in the plasma, and other SM molecules found in the ectoplasmic leaflet of the plasma membrane. They are thus thought to play specific signaling roles [37].

All complex SLs can be degraded to produce Cer, which is then converted to Sph via the action of ceramidases [22]. Ceramidases have an organelle-specific expression, allowing the cell to generate distinct SLs with certain sphingoid bases [22]. Like SMases, ceramidases can be classified according to their pH optima. Whereas acid ceramidase is required for the lysosomal degradation of Cer, neutral ceramidases are necessary for sphingosine 1-phosphate (S1P)-mediated signaling on the plasma membrane. On the tissue level, neutral ceramidases are required for the breakdown of dietary SLs [38, 39]. Alkaline ceramidases work near the plasma membrane. After Cer is deacylated by any of the ceramidases, Sph can be converted to S1P through the action of two sphingosine kinases distributed in the cytosol, and other membrane compartments [40–43]. Different isoforms of the alkaline ceramidase family (alkaline ceramidases 1, 2, and 3) are required to maintain high blood levels of S1P in mice [44]. Alkaline ceramidase 2 is specifically necessary to regulate the plasma pools of S1P and sphinganine 1-phosphate [45]. Finally, S1P is degraded by S1P lyase to produce hexadecenal and phosphoethanolamine [22].

Deficiencies in the hydrolases and other ancillary proteins involved in GSL, SM, and Cer degradation lead to the development of sphingolipidoses. About ten different disorders are caused by such deficiencies [46].

## SL-related LSDs: Sphingolipidoses

**Fig. 3:**
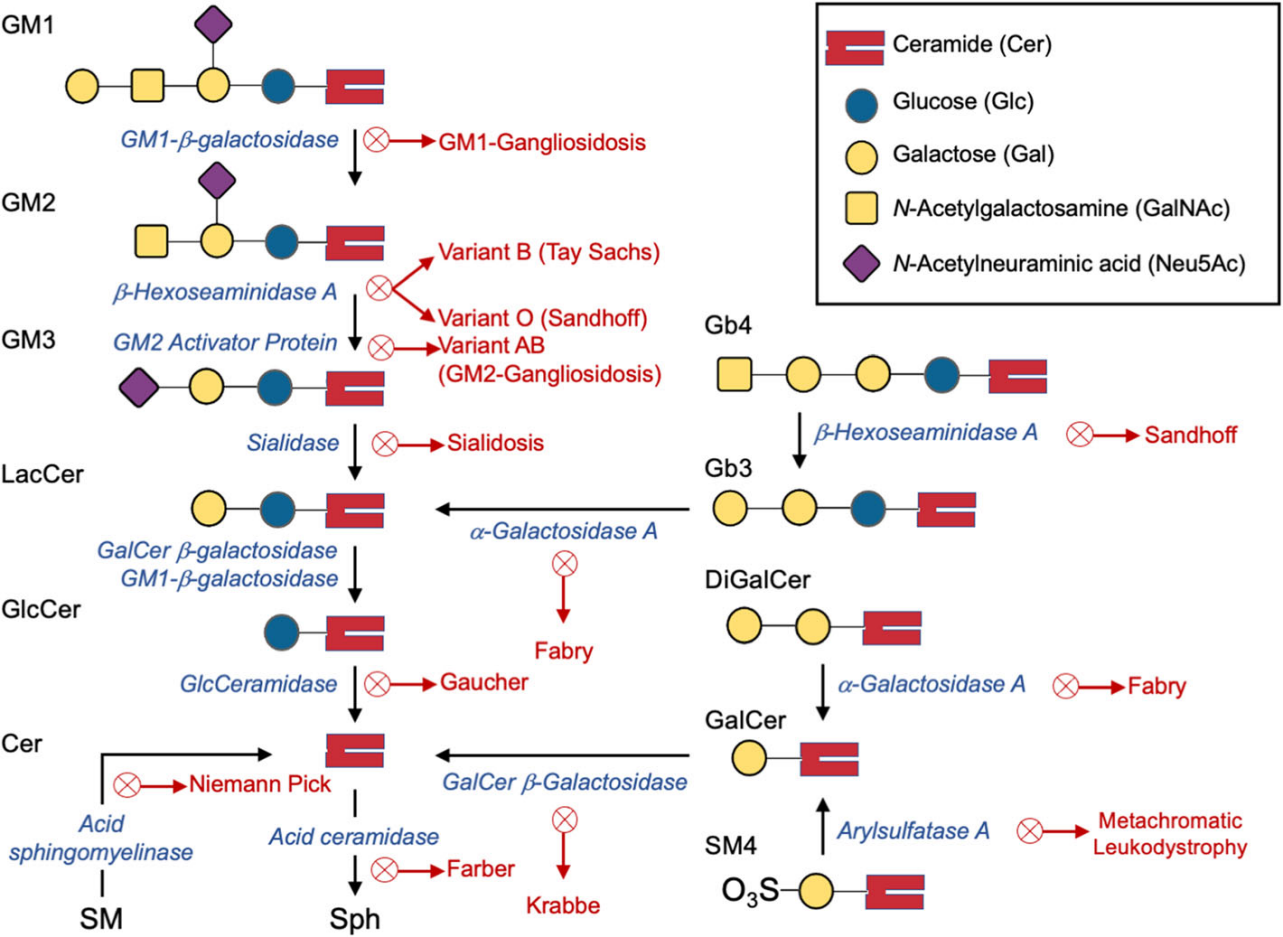
Lysosomal SL catabolism and enzyme deficiencies causing storage diseases. A schematic of the various SL metabolic pathways is presented, indicating the enzymes whose deficiency leads to several diseases. Note that each enzyme is assisted by one or more Saps: GM2A assists both β-gal and β-hexosaminidase. SapB assists sialidase, α-GAL, GALC, and β-gal. SapC assists GALC, β-gal, GCase, acid ceramidase, and GALC. Acid ceramidase is also assisted by SapD

SLs are catabolized in a strictly sequential manner. Defects in the machinery controlling each step of the pathway are prevalent, and several diseases have been described (Fig. 3). Most SLs are degraded in the lysosome via a single pathway; a deficiency in one enzyme will lead to the accumulation of the molecule to be catabolized. An exception to this pattern is lactosylceramide (LacCer), which can be degraded by two different lysosomal enzyme/ activator protein systems; thus, it does not accumulate solely by a deficit in a single enzyme [46]. Nevertheless, LacCer can accumulate along with other substances, when multiple factors are absent (*e.g.,* prosaposin) [30].

To allow for a better understanding of each disease, genes of most deficient enzymes leading to sphingolipidoses have been cloned and targeted in animal models [47]. Despite being sub-classified into types differing in the onset, severity, and associated tissues, each sphingolipidosis has a clinical continuum of severity (Table 1).

**Table 1:** Summary of the Forms and Symptoms of Sphingolipidoses

LSD	Defective enzyme	Mutated gene	Major accumulating SL(s)	Onset	Symptoms and neurological manifestations	Refs.
**GM1 Gangliosidosis**	β-gal	*GLB1*	GM1	Type I: First year Premature death at age 2-3	• Developmental arrest • Seizures • Disintegration in the nervous system • Stiffening of joints • Hepatosplenomegaly • Edema • Gum hypertrophy • Skeletal abnormalities • Cherry-red spot (50% of the population) • Corneal cloudiness followed by blindness and deafness	[46, 48–52]
				Type II-late infantile: 7 months-3 years	• Developmental delay • Subsequent dementia • Cerebellar pyramidal, and extrapyramidal signs • Possible late loss of vision • No skeletal dysplasia	
				Type III:3-30 years	• Dysarthia and gait disturbances • Dystonia in the neck and extremities • Extrapyramidal signs • Cardiomyopathy	
**Sandhoff (Variant B)**	α-subunit of β-Hexoseaminidase	*HEXA*	GM2, lyso-GM2	Infantile (Tay-Sachs): 3-6 months	• Loss of skills • General weakness • Seizures • Bone abnormalities • Cherry-red spot • Startle response • Demyelination and swelling of neuronal cells • Reduction of consciousness, vision, and hearing. • Eventual spasticity and death.	[46, 53–57]
				Juvenile: 2-6 years with death at 10-15 years	• Progressive spasticity • Loss of speech and vision • Progressive dementia • Infertility	
				Chronic: 2-5 years but patients can reach their fourth decade	• Chronic: Gait disturbances • Posture abnormalities, followed by distinct neurological symptoms. • No sensory or intellectual impairment • Adult: has heterogeneous symptoms with intact mental and visual capabilities. • Bipolar psychosis may develop	
**Sandhoff (Variant O)**	β-subunit of β-Hexoseaminidase	*HEXB*	GM2, lyso-GM2, uncharged glycolipids like GA2	Infantile: 6 months	• Same as Tay-Sachs with fewer bone deformities, and organomegaly.	[46, 58, 59]
				Juvenile: 2-10 years	• Cerebellar ataxia • Slurred speech • Psychomotor retardation followed by gradual mental retardation • Spasticity	
				Adult: in late adult life	• Pyramidal and extrapyramidal signs and symptoms of lower motor neurons • Supranuclear ophthalmoplegia • Movement problems	
**Sandhoff (Variant AB)**	GM2A protein	*GM2A*	GM2, GA2	3-6 months	• Muscle weakening. • Loss of motor skills (crawling and sitting) • Startle reaction to noises • Seizures • Loss of vision and hearing • Intellectual disability • Paralysis	[60–63]
**Gaucher Disease**	GCase	*GBA1*	GlcCer, GlcSph	Type I (Non-neuronopathic): Infancy to late adulthood	• Massive abdominal distension • Anemia and thrombocytopenia • Defective platelet function (abnormal coagulation) • Organomegaly • Poor development and delayed puberty • Bone diseases • Hepatopulmonary syndrome • No neurologic symptoms	[46, 64–71]
				Type II: 3-6 months with death at ~2 years	• Collodion skin • Visceral and bone marrow involvement • More severe neurological manifestations: Strabismus • Fast eye movement. • Bulbar palsy or paresis. • Severe hypertonia, rigidity, arching, swallowing impairment. • Seizures. • Progressive dementia. • Ataxia	
				Type III: 2-5 years	• Visceral and bone marrow involvement • Less severe neurological manifestations with slower progression	
**Niemann Pick A, B**	aSMase	*SMPD1*	SM	NPD-A: early onset premature death at the age of 3	• Lymphadenopathy • Hepatosplenomegaly Hypotonia • Muscular weakness leading to feeding difficulties, followed by decreased platelet count, microcytic anemia • Osteoporosis • Cherry-red spots in the eye • Brownish-yellow color of skin • After six months of age, psychomotor retardation is observed • Loss of contact with the surroundings	[46, 72, 73] [74–77]
				NPD-B: chronic ranges from infancy-adulthood	• Slowly progressive systemic symptoms • No neurodegeneration • Hepatosplenomegaly • Anemia • Thrombocytopenia • Liver dysfunction • Lung and bone diseases.	
**Farber Disease**	Acid Ceramidase	*ASAH1*	Cer	Type I: Early-onset premature death at age 2-3 years	• Hepatosplenomegaly • Joint contractures • Voice hoarseness • Inflammation of subcutaneous nodules, along with other neurological manifestations	[46, 78–81]
				Type II: intermediate	• Decreased neurological inflammation-related symptoms • Longer lifespan	
				Type III: mild		
				Type IV: Neonatal-visceral	• Organomegaly and visceral manifestations	
				Type V: Neurological-Progressive	• Progressive neurodegeneration and seizures	
				Type VI	• Combined Farber and Sandhoff diseases and associated symptoms	
**Fabry Disease**	α-GAL	*GLA*	Gb3, lyso-Gb3	**Males**: During childhood or adolescence	• Corneal dystrophy • Acroparesthesia • Angiokeratomas, and hypohidrosis, followed by progressive multi-system involvement leading to kidney failure, cerebrovascular disease, and hypertrophic cardiomyopathy in affected males • Females range from having no symptoms to severe ones.	[82–89]
				**Females**: Heterozygous Mild late-onset disease (adult-onset) or severe disease. Homozygous Similar onset as males		
**Krabbe Disease**	GALC	*GALC*	psychosine	Infantile: 3-6 months. premature death between 2-5 years of age	• Motor dysfunction • Seizures • Cognitive decline	[46, 67, 90–92]
				Juvenile & adult: few years- 73 years	• Dementia • Blindness, Psychomotor retardation • Spastic paraparesis	
**Metachromatic Leukodystrophy**	ASA	*ASA*	sulfatide	Late infantile: Before 30 months	• Hypotonia • Mental regression, Unsteady gait, followed by loss of speech • Incontinence • Blindness • Seizures • Peripheral neuropathy • Complete loss of motor function • Loss of contact with the surroundings is observed before reaching 40 months of age	[46, 93–95]
				Juvenile: 2.5-16 years	• Later in onset but once the ability of walking is lost, the disease progresses as seen in the infantile form • Infertile	
				Adult: After puberty	• Variable progression	
**Niemann Pick C1**	NPC1	*NPC1*	Chol and other SLs	Perinatal (up to 2 months)	**Systemic:** • Mild thrombocytopenia (newborns or toddlers) • Prolonged neonatal cholestatic jaundice (in perinatal) • Hepatomegaly/ Splenomegaly **Neurological:** • Vertical supranuclear • Gaze palsy • Gelastic cataplexy • Ataxia • Dystonia • Dysarthria • Dysphagia • Hypotonia • Clumsiness • Delayed developmental milestones • Seizures • Hearing loss **Psychiatric** • Psychosis • Cognitive decline • Developmental delay	[96–99]
**Niemann Pick C2**	NPC2	*NPC2*				
				Early-infantile (2 months–2 years of age)		
				Late-infantile (2–6 years of age)		
				Juvenile (6–12 years of age)		
				Adolescent/adult (>12 years of age)		
**Sialidosis**	Sialidase (Neuraminidase)	*NEU1*	sialyloligosaccharides	Sialidosis type I: Second to third decade	• Macular cherry-red spot • Gait abnormalities • Decreased visual acuity • Normal to slightly impaired intelligence • Action myoclonus • Intentional tremors • Cerebellar ataxia • Hyperreflexia • Hypotonia may occur • Cerebellar atrophy in advanced stages	[100]
				Sialidosis type II-congenital hydropic: in utero	• Hydrops fetalis: Ascites, Edema • Hepatosplenomegaly • Course features • Stillbirths or death at a very early age • Inguinal hernia • Cardiac Abnormalities • Renal Abnormalities • Respiratory distress • Psychomotor retardation • Hydrocephalus • Seizures • Corneal clouding • Dysostosis multiplex	
				Sialidosis type II-infantile: 0–12 months	• Coarse features • Hepatosplenomegaly • DysostosisMultiplex • Cherry red spot • Corneal Clouding • Cataract • Hearing loss • Inguinal hernia • Umbilical hernia • Hypotonia	
				Sialidosis type II- juvenile: 2–20 years	• Psychomotor delay • Seizures • myoclonic jerks • Ataxia • Myoclonic epilepsy	

### GM1 Gangliosidosis

The lysosomal hydrolase GM1-β-galactosidase (β-gal) is assisted by either SapB or GM2A to catalyze the breakdown of GM1 ganglioside to GM2 [46]. A defect in such an enzyme may lead to GM1 gangliosidosis, which is an autosomal recessive and neurodegenerative disease with an estimated incidence of 1 in 100,000–200,000 live births [101]. Another disorder, Morquio syndrome type B, may also develop depending on the substrate specificity of the defective enzyme [46, 101].

Any mutation in the *GLB1* gene leading to reduced or loss of activity of β-gal causes the accumulation of lysosomal GM1 [48]. Depending on the specific *GLB1* mutation, the residual activity of β-gal differs, leading to a continuum of clinical severity. GM1 gangliosidosis can be classified into three types: Infantile (Type I), late infantile/ juvenile (Type II), and adult (Type III). Also, Type II can be subdivided further into late-infantile (IIa) and juvenile (IIb) [102] (Table 1).

Although GM1 is crucial for many neuroprotective purposes [103], its massive lysosomal accumulation stimulates neuroinflammatory reactions and the unfolded protein response (UPR) in mouse models of the disease, leading to neuronal death and neurodegeneration [46]. Although no cure for the disease currently available, chaperone therapy [104], substrate reduction therapy (SRT) [105], and gene therapy [102] have been shown to reduce the storage levels of GM1 in the brains of mouse models (see later).

### GM2 Gangliosidosis

GM2 gangliosidoses are autosomal recessive, neurodegenerative diseases caused by defects in the machinery responsible for GM2 degradation, leading to the accumulation of GM2 and other related lipids in neural cells [106]. Normally, GM2 is degraded by the coordinated action of the lysosomal β-*N*-acetyl-hexosaminidase (β-hexosaminidase), which removes the terminal *N*-acetyl-galactosamine residue from GM2, and the ancillary protein GM2A. β-hexosaminidase has two hydrolytic subunits (α, and β) whose different combinations may form three distinct isozymes, with different substrate specificities. HexA (αβ) cleaves off terminal *N*-acetylglucosamine and *N*-acetylgalactosamine residues linked to uncharged and negatively charged glycoconjugates like GM2, whereas HexB (ββ) is more specific to uncharged substrates like glycolipid GA2 [46, 53]. HexS (αα) is a secondary type that contributes to the degradation of sulfated glycolipids, and glycosaminoglycans [107]. A defect in any of the components comprising the GM2 degradation machinery leads to a different type of GM2 gangliosidosis: variant B (α-subunit deficiency, Tay-Sachs in its infantile form), variant O (β-subunit deficiency, Sandhoff disease), and variant AB (GM2A deficiency) (Table 1).

A special variant (B1) has an altered enzymatic specificity of HexA. Though it has no activity towards negatively-charged substrates, including GM2, its activity remains intact towards uncharged substrates [108]. This is attributed to the conservation of the β-subunit activity, subunit association, and enzyme processing, although the active site of the α-subunit is defective [109]. The symptoms of B1 variant patients resemble those of the juvenile form of B variant. However, heterozygotes of B1 and null alleles show the late-infantile course of the disease [46].

In the O-variant/ Sandhoff disease, the storage of negatively-charged glycolipids that characterize Tay-Sachs disease is accompanied by the storage of other uncharged glycolipids like GA2 in the brain and other visceral organs (Table 1) [46, 58]. A similar picture of Tay-Sachs disease with a delayed onset can be observed in patients with normal β-hexosaminidase A, B, and S isozymes. These AB-variant patients have a deficient GM2A, leading to the accumulation of GM2 and GA2 [60].

Generally, GM2-gangliosidoses and their accumulated compounds (GM2, GA2, and cytotoxic lyso-GM2) cause neuroinflammation and other secondary effects, leading to swollen demyelinated neurons of mainly the central and also the peripheral nervous system in humans and model animals [46]. Thus, multiple therapeutic strategies, including SRT [64] and gene therapy [18], have been suggested to decrease the number of accumulated lipids.

### Gaucher Disease

Gaucher disease (GD) is the most common autosomal recessive sphingolipidosis [46], with an incidence ranging from 0.39 to 5.80 per 100,000 in the general population [110]. GD can be classified into three major types: Type I GD (non-neuronopathic), Type II GD (neuronopathic acute form), and Type III GD or the juvenile form (neuronopathic sub-acute) (Table 1). Type I GD has a higher prevalence (1 per 850) in Ashkenazi Jews as compared to 1-2 per 100,000 in non-Jewish populations [110].

Mutations in the *GBA1* gene that encodes glucosylceramide-β-glucosidase (GCase) lead to the accumulation of GlcCer. GCase normally works in coordination with SapC and lysosomal BMP to hydrolyze GlcCer into glucose and Cer. Therefore, in rare circumstances, GD can also be caused by a deficiency in SapC [65, 111]. The reduced cellular capacity to degrade GSL leads to the primary accumulation of GlcCer in cells, particularly phagocytizing macrophages mainly found in the liver, spleen, and bone marrow. This leads to the development of storage macrophages called “Gaucher cells” that characterize the disease [64]. GlcCer is further metabolized through the action of lysosomal acid ceramidase to produce a secondary storage substance, glucosylsphingosine (GlcSph), which can exit the lysosomal compartment [66, 112]. Accumulated GlcCer and GlcSph in the cytosol can be further hydrolyzed by non-lysosomal GCase-2 to produce Cer, Sph, and S1P [113, 114]. Although these events were shown to occur peripherally, their occurrence in the brain is not clear [115].

### Krabbe’s Disease

Globoid cell leukodystrophy, or Krabbe disease (KD), is another autosomal recessive, neurodegenerative disease that is characterized by a defective galactosylceramide β-galactosidase (GALC) [67]. GALC uses the help of SapA and SapC [46] to remove galactose from its primary substrate GalCer and other secondary galactose-containing SLs, *e.g.*, galactosylsphingosine (psychosine) [67]. The primary substrate does not accumulate in the central nervous system (CNS), because it can be degraded by another hydrolytic system (β-gal) [116]. Instead, psychosine is the major accumulating product. Psychosine is a cytotoxic substance that causes demyelination by triggering the disintegration of oligodendrocytes and Schwann cells, the myelin-forming cells in the central and peripheral nervous system, respectively [67]. Besides demyelination, KD causes infiltration of large, multinucleated macrophages, and perivascular microglia [117] forming “globoid cells” engorged with undigested storage SLs in the white matter. This is accompanied by astrogliosis and pro-inflammatory cytokine dysregulation [90]. There are different forms of KD: infantile-, juvenile- and adult-onset (Table 1) [46, 90].

A mimicry of the course of KD is achieved in the twitcher mouse, in which a premature stop codon in the coding region of the *GALC* gene was engineered [118]. Other mouse models were modified to show low levels of residual activity [119]. Such murine models can be utilized for stem cell transplantation and other therapeutic strategies, to target multiple pathogenic pathways as a means to reduce progression of the disease [90].

### Fabry Disease

Fabry disease is a pan-ethnic, X-linked genetic disorder with an approximate incidence of about 1 per 117,000 live births in the general population [120], and 1 per 40,000 male live births [121]. It is caused by a deficiency in α-galactosidase A (α-GAL), leading to the accumulation of Gb3 and other related SLs in multiple cells [82]. Globotriaosylsphingosine (lyso-Gb3) is a deacylated form of Gb3, and it forms the secondary storage metabolite that is used as a biomarker of the disease, accumulating to high levels in vasoendothelial cells [83]. Lyso-Gb3 was found to play roles in nephropathy and secondary inflammatory events [122, 123]. There are two major types of Fabry disease: infantile and late-onset forms [84] (Table 1).

### Metachromatic Leukodystrophy

Metachromatic Leukodystrophy (MLD) is an autosomal recessive LSD, with an incidence of 1 per 40,000-160,000 live births. It is caused by mutations in the gene encoding arylsulfatase A (ASA) [93]. ASA, which is assisted by SapB [46], catalyzes the conversion of *O*-sulfogalactosylceramide into GalCer and sulfate [94]. MLD is characterized by the accumulation of sulfatides and other related glycolipids in the lysosome. Because sulfatides are present mainly in the white matter of the brain and peripheral nervous system (PNS), forming the myelin sheath, sulfatide accumulation causes predominantly demyelination. Secondarily, a cytotoxic sulfatide derivative, lyso-sulfatide, is thought to play a role in the pathogenesis of the disease [46]. Based on the age of onset, three forms of MLD can be identified: late infantile, juvenile, and adult [94] (Table 1). A similar clinical picture of MLD is observed in patients with SapB deficiency [46].

### Niemann Pick Disease (Types A, B, & C)

Niemann Pick disease types A and B (NPD-A and B) are autosomal recessive LSDs, with an estimated prevalence of 0.4–0.6 per 100,000 [124]. They are caused by the deficiency of aSMase, leading to the accumulation of SM within several cell types, including hepatocytes, macrophages, reticuloendothelial cells, and neurons [72, 74]. Accumulation of SM and related SLs in the monocyte/ macrophage system forms the so-called “foam cells” that characterize the disease [46]. Clinically, the symptomatic spectrum of NPD ranges from extremely severe to relatively mild. Neurovisceral NPD-A is the most severe form, whereas NPD-B (the chronic visceral form) is on the other end of the spectrum [74] (Table 1).

In the absence of an aSMase deficiency, another type of NPD can still develop (NPC), which is another autosomal recessive neurodegenerative disease with an incidence of about 1 per 120,000 live births. It is caused by mutations in the *NPC1* and *NPC2* genes that encode Chol-transporting proteins (Table 1). NPC is characterized by the accumulation of Chol and SLs [125]. Whereas NPC1 protein is required for the retrograde fusion of lysosomes with endosomes to form hybrid organelles [96, 126], NPC2 is involved in membrane fission events to regenerate lysosomes from hybrid organelles [127]. Defects in them lead to the accumulation of unesterified Chol, SM, GSLs, and Sph [96]. This results in the disruption of endocytosis, the vesicular fusion between late endosomes and lysosomes [97], and calcium ion homeostasis in multiple cells. Neuronal disruption of these events leads to dementia, loss of cerebellar Purkinje neurons, epilepsy, ataxia, and vertical gaze paralysis.

### Farber’s Disease

Farber’s lipogranulomatosis (or disease) is an extremely rare autosomal recessive LSD caused by mutations in the *ASAH1* gene that expresses acid ceramidase. Acid ceramidase hydrolyzes ceramide with the assistance of SapC or SapD [46]. Enzyme deficiency leads to Cer accumulation [78]. Farber’s disease is classified into different subtypes: Type I patients exhibit severe neurological manifestations culminating in premature death at age 2-3 years [78, 79]. Patients with types II and III have decreased neurological involvement and longer lifespan, and are therefore termed “intermediate” and “mild” forms, respectively, although they do show inflammation-related symptoms [78]. Types IV and V are termed “Neonatal-visceral,” and “Neurological-Progressive” [78, 80]. Finally, prosaposin deficiency, in which the precursor of all Saps is deficient, may show some clinical manifestations similar to those of Farber’s [78].

## Pathophysiology of Sphingolipidoses

Sphingolipidosis pathogenesis is a network of multiple affecting mechanisms, beginning with the accumulation of the primary substrate(s) of the deficient enzyme, then spreading to other compartments and progressing to other secondary effects/ deficiencies, and ultimately leading to an intricate pattern of defective storage [128]. The primary cellular response to any LSD is the production of more lysosomes, but because these organelles are deficient in the same enzyme, the newly formed lysosomes will be abnormal as well, resulting in a halt in the lysosomal system. This halt is responsible for endocytic, autophagic, and inflammatory abnormalities eventually causing cellular death [129]. Common factors influencing the pathogenesis of sphingolipidoses are presented hereafter.

### Cell-Type-Specific Patterns

The observed heterogeneity in affected organs within sphingolipidoses is attributed to cell-type-specific glycolipid localization. Lipid storage and its associated pathogenesis occurs in tissues in which the accumulating lipid is either generated predominantly or endocytosed [46, 129]. For example, the neural dysfunction observed in GM1 and GM2 gangliosidoses [9] is due to the abundance of sialic acid-containing GSLs (especially GM1 and GM2) in the brain, particularly on the surfaces of nerve cells [130, 131]. In GD, however, the primary accumulation of GlcCer is in macrophages. Macrophages phagocytize other cells, consolidating large amounts of accumulating GlcCer, and directly causing pathogenesis in phagocytic cells [132]. Additionally, because the ratio of Cer to GlcCer is important in maintaining the epidermal permeability barrier [133], many GD patients experience ichthyotic, dry skin due to abnormal transepidermal water loss [134]. On the other hand, in MLD and KD, the major pathological manifestations are severe demyelination and neurodegeneration [135]. These are attributed to the high abundance and importance of sulfatides and GalCer in glycosynapses, myelination, and oligodendrocyte function [136].

### Residual Activity

In LSDs, enzymes may be completely or partially deficient, leading to some remaining (residual) activity. An improperly-folded enzyme cannot reach the lysosome and is degraded in the ER, resulting in a complete loss of activity [137], whereas a less-active mutant enzyme that can reach the lysosome may contribute to a degree of residual activity [138]. The diversity in the onset and severity of the disease is determined by the residual activity of the dysfunctional lysosomal enzyme. A more severe, early-onset course of a disease results from a complete deficiency/ extremely low activity of the enzyme, whereas a delayed, milder form can be due to a slight increase in the degree of residual activity [129, 139]. Nonetheless, a patient's phenotype cannot be predicted precisely based on this simple correlation. Biochemical evaluation of the mutated enzyme will be required to determine the molecular basis for the development of the disorder [140]. Further, other epigenetic factors may result in phenotypic variability between patients carrying the same mutant alleles [141].

Low residual activity below a certain threshold can cause substrate accumulation and a subsequent pathological phenotype [142]. The ‘threshold theory’ may explain the pseudo-deficiency phenomenon in which a patient may carry a defective enzyme yet still show a normal phenotype, with no substrate accumulation, thus indicating the presence of an above-threshold activity of the enzyme [129]. It also explains why some slight changes in residual activity can ameliorate significantly the symptoms. This theory and its associated explanations aided the development of the chaperone therapy as a therapeutic approach to many sphingolipidoses [143].

### Nature of Accumulating Storage Materials

The nature of the storage material is a major contributor to the pathogenesis of LSDs, as it may result in the accumulation of other bioactive molecules [46].

Psychosine, which can destabilize membranes due to its detergent-like properties [67], accumulates in cells of KD patients. Endogenous psychosine is synthesized by Cer galactosyltransferase (CGT), predominantly expressed in the third stage of oligodendrocyte differentiation [67] and the Schwann cell myelinating process [144]. Normally, GALC maintains low levels of brain psychosine, but under GALC-deficient conditions, psychosine accumulates to make up about 50% of brain cerebrosides [145]. Psychosine accumulation disrupts lipid raft architecture, leading to dysregulation of some signaling pathways. Psychosine-induced inhibition of protein kinase C (PKC), which normally activates Schwann cell proliferation in PNS [145, 146], causes synaptic dysfunction, demyelination, and axonal defects. In the CNS, both exogenous and endogenous psychosine cause oligodendrocyte cell-body atrophy and apoptosis [67]. Psychosine also induces cell death via the activation of the secretory phospholipase A2, which produces lysophosphatidylcholine and arachidonic acid that lead to oligodendrocyte death [147]. Psychosine also inhibits the oligodendrocyte survival-signaling pathways Akt and ERK [148, 149]. Moreover, even if some oligodendrocytes survive psychosine toxicity during differentiation, psychosine inhibits oligodendrocyte peroxisomal function by inhibiting the expression PPARα that normally induces the expression of other peroxisomal proteins, DHAP-AT and PEX11, which are responsible for myelin formation and maintenance [67]. Therefore, psychosine contributes to the pathogenesis of KD in the CNS by impeding normal oligodendrocyte differentiation and subsequent maturation leading to demyelination. Nevertheless, the complex neurological dysfunction observed in KD patients is not due to demyelination alone. Rather, it is a combination of demyelination and fast axonal transport inhibition. Psychosine accumulation blocks fast axonal transport by stimulating axonal GSK3β and PP1, altering their interaction with membrane rafts. These proteins abnormally phosphorylate and inhibit kinesin light chain, thus inhibiting the activity of the motor protein required for fast axonal transport [150]. Additionally, microglial cells are also affected by psychosine accumulation after phagocytizing myelin aggregates and damaged oligodendrocytes. Psychosine appears to inhibit cytokinesis in the microglial cell cycle, resulting in the formation of multinucleated globoid cells that characterize KD via an unknown inhibition pathway [151, 152].

Unlike psychosine, much less is known about the effects of lysosulfatide, lyso-GM1, and lyso-GM2. Lysosulfatide is a cytotoxic compound that accumulates in the brains of MLD patients and ASA-deficient mice. It was suggested that lysosulfatide contributes to disease pathology by lipid raft disruption [153]. Lyso-GM1 and lyso-GM2 accumulate in GM1 and GM2 gangliosidoses, respectively. Although the exact mechanisms by which they contribute to pathogenesis are still unknown, they inhibit PKC [152, 154].

GlcSph is another cytotoxic material elevated in the brains of GD type 2 and 3 patients. GlcSph together with GlcCer, hexosylsphingosine, and BMP, and the associated altered SL/ Chol content, contribute to the disruption of membrane raft architecture, thereby impairing cell signaling, calcium homeostasis, and resulting in other secondary effects [155, 156].

Other secondary metabolites that are unrelated to the defective enzyme may also accumulate in LSD cells. In NPC patients, for instance, secondary storage of GM2 and GM3 is caused by defects in trafficking and lysosomal calcium ion homeostasis. Although not completely understood, Chol accumulation in many sphingolipidoses is also caused by defects in lipid trafficking [157, 158].

Another interesting feature of various LSDs is the accumulation of ɑ-synuclein, a protein that characterizes Parkinson’s disease, and is usually found in the presynaptic termini of brain neurons [159, 160]. ɑ-synuclein oligomers are found in GD, KD, and NPC patients [161]. It may also aggregate with other lipids to form Lewy bodies that were found in brain samples of GD and GM2 gangliosidosis patients [162]. ɑ-synuclein aggregates might participate in pathogenesis via multiple secondary effects, including altered calcium ion homeostasis [163], inhibited autophagy [164], and disrupted mitochondrial function [162].

### Secondary Effects

#### Inflammation and Cytokine Release

One of the first innate immune responses against infection, injury, or damage is the acute inflammatory response. It is initiated by immune cells when they recognize damage-associated molecular patterns released from injured or dying cells [165, 166]. The response involves the release of inflammatory cytokines that result in leukocyte migration into tissues. The normal acute inflammation stops once the trigger disappears, whereas chronic systemic inflammation involves the continuous activation of the inflammatory response, resulting in attacks on neighboring cells, and causing their death [167].

The role of inflammation in the pathogenesis of sphingolipidoses was first established in GD patients and models. GD storage substrates, GlcCer and GlcSph, accumulate mainly in macrophages, resulting in their abnormal activation [128]. Dysfunctional macrophages activate their inflammasome due to the impaired autophagic process, which leads to an unregulated secretion of interleukin 1β (IL-1β). Furthermore, the levels of several other cytokines including tumor necrosis factor α (TNF-ɑ) and chitotriosidase are elevated in the plasma of GD patients [168, 169]. These mediators recruit other immune cells, including other macrophages and neutrophils, to the site of inflammation. Because these cells carry the mutation, however, their arrival amplifies the disease [128]. Moreover, GD patients suffer from increased immunoglobulin production (known as gammopathy). Monoclonal gammopathies result in increased susceptibility to myeloid cancer through increased levels of IL-6 and IL-10. IL-6 contributes to an expansion of myeloid cells, while IL-10 results in the production of autoantibodies and B-cell lymphomas [128, 170]. M2 macrophage activation may link GD to cancer, though the mechanism is not fully understood [65, 171].

In neuronopathic GD mouse models, increased levels of the macrophage colony-stimulating factor, TNF-ɑ, IL-1β, and TGF-β contribute to neuroinflammation [168, 172]. This cytokine release is linked with microglial activation that results in neuronal cell death [173]. Mechanistically, once GlcCer levels surpass a specific threshold, neurons trigger signaling cascades that result in microglial activation. Microglial activation induces a neuroinflammatory cascade leading to the release of cytokines and reactive oxygen species (ROS), as well as the increased permeabilization of the blood-brain barrier (BBB) [174]. These events lead to chronic neuroinflammation that ultimately leads to neuronal apoptosis through receptor-mediated caspase activation, followed by caspase-dependent- and independent- activation of mitochondrial cell death. Microglial activation followed by neuronal cell death is also observed in GM1 and GM2 mouse models [174].

Pathogenesis of Fabry disease is also partly caused by inflammation [128]. Fabry disease patients have high nitric oxide and lipid peroxidation levels, as well as abnormal glutathione metabolism indicative of enhanced ROS production [175]. ROS-induced oxidative protein damage contributes to the generation of neoantigens that induce autoimmune disorders [174]. Studies on Fabry disease knockout mice showed that Gb3 storage leads to disruption of the CD1 antigen-presentation pathway, and invariant natural killer T cell distribution [176]. Gb3 and lyso-Gb3 also induce the constitutive secretion of proinflammatory cytokines via the Toll-like receptor 4 (TLR4)-mediated pathway [128].

Inflammation is also implicated in Farber disease pathogenesis. In knock-in mouse models, Cer accumulation results in an early elevation of multiple proinflammatory cytokines, mainly monocyte chemoattractant protein 1, which cause the formation of subcutaneous nodules and other pathological manifestations characterizing the disease [177].

Although inflammation is a secondary effect resulting from downstream cascades in sphingolipidoses, it might be targeted in LSD therapeutic approaches to alleviate inflammatory symptoms. Non-steroidal anti-inflammatory drugs (NSAIDs), for instance, were used to treat Sandhoff mice models with elevated levels of the macrophage inflammatory protein ɑ, thus preventing the recruitment of immune cells to the brain and the subsequent neuroinflammation [178]. NSAIDs were also used in treating NPC1 mouse models [179].

#### Calcium Ion Homeostasis

**Fig. 4:**
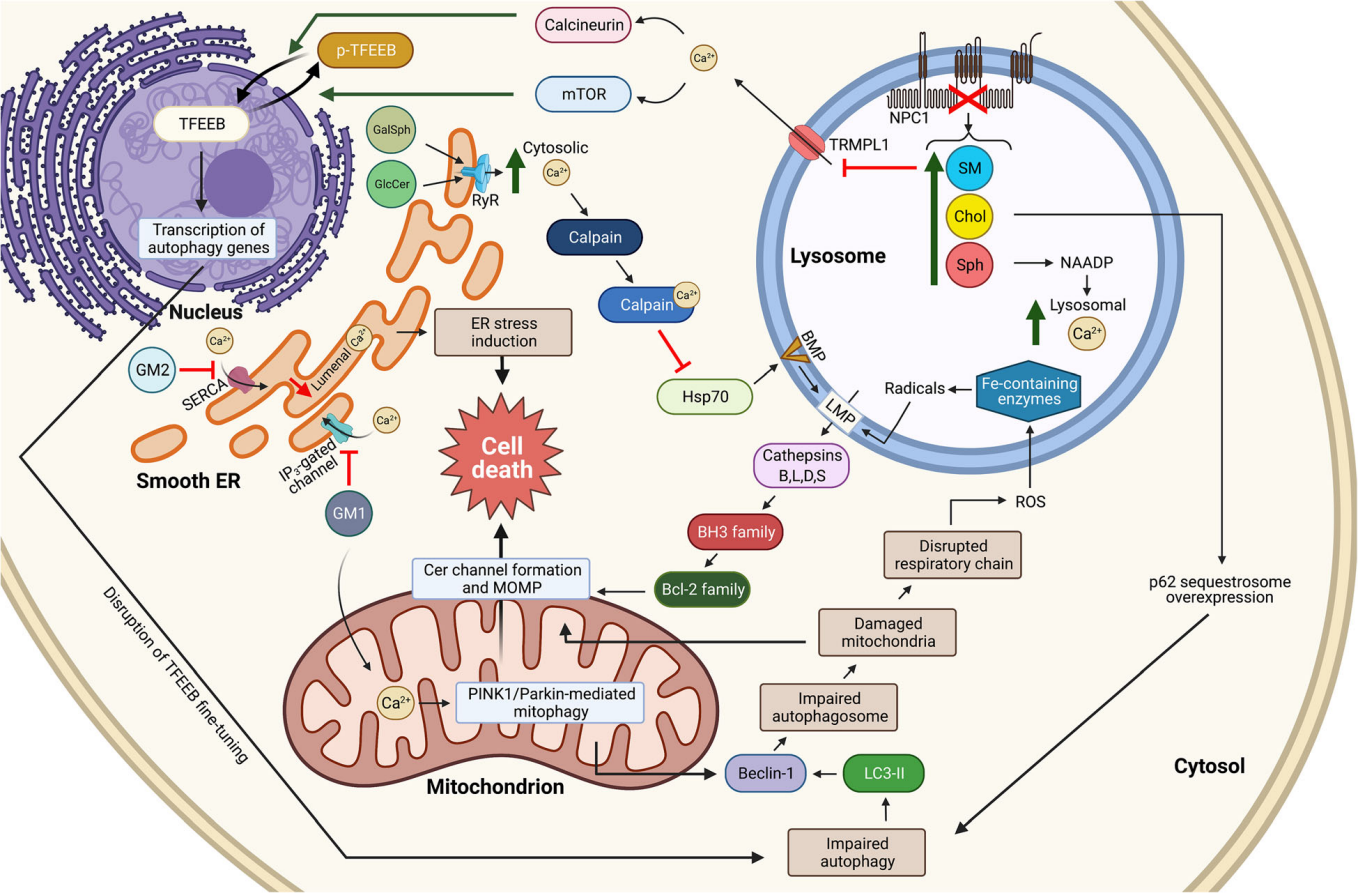
Summary of the major cellular interactions leading to the neurological features of sphingolipidoses. A schematic of the main events that lead to caspase-dependent and independent activation of neuronal apoptosis through myriad intercalated pathways, such as the disruption of TFEEB fine-tuning, impaired Ca^2+^ homeostasis in smooth ER, mitochondria and lysosomes, lysosomal membrane permeabilization, and impaired autophagy, along with others (not depicted here). Proteins are depicted in oblong shapes, while lipids are in circles and cellular events are in rectangles. Upward green arrows represent increase in cellular concentration while downward red arrows illustrate decreased concentration. Note that organellar sizes are not to scale. not to scale. This figure was created in Biorender.com

Calcium is a crucial factor in the regulation of myriad cellular events. An intracellular defect that leads to impaired Ca^2+^ homeostasis will lead to ER and oxidative stress, and eventually cell death. The mechanisms by which impaired Ca^2+^ homeostasis occurs can be variable among different LSDs, depending on the type of interaction between the storage material and specific Ca^2+^ pumps or channels in different organelles [180]. Depending on the defective organelle, Ca^2+^ homeostasis can be classified as altered ER, mitochondrial, or lysosomal function (Fig. 4).

Altered ER Ca^2+^ homeostasis can be observed in the neuronal forms of GD and KD, in which increased ER Ca^2+^ release occurs due to direct modulation of the ryanodine receptor by GlcCer and psychosine [180, 181]. In Sandhoff disease [182] and NPD-A disease [183], cytosolic Ca^2+^ uptake into the sarcoplasmic reticulum by the Sarco/ endoplasmic reticulum Ca^2+^-ATPase (SERCA) is decreased [182]. In Sandhoff disease, uptake reduction is attributed to the modulation of SERCA activity by the protruding sialic acid part of the ganglioside GM2 [184]. Sarcoplasmic reticulum Ca^2+^ stores are also depleted in GM1 gangliosidosis through the interaction of GM1 with the phosphorylated inositol triphosphate-gated Ca^2+^ channel [185]. Moreover, GM1, GM2, and GM3 interact with, and reduce the activity of the plasma membrane Ca^2+^-ATPase (PMCA), which pumps cytosolic Ca^2+^ into the extracellular space [186]. In liposomes containing high SM, PMCA activity is diminished, possibly through SM interference with the proper folding of PMCA, or through alteration in raft compartmentalization, thus altering its interaction with other binding proteins [187].

Mitochondria are strongly engaged in Ca^2+^ signaling, both by providing the energy required for its transport, and by directly participating in its signaling events. A defect in Ca^2+^ homeostasis will cause severe mitochondrial damage in at least two sphingolipidoses [162]. GM1 accumulation in mouse embryonic fibroblasts (MEFs) in β-gal-deficient mice increases mitochondrial Ca^2+^ load, leading to the stimulation of the mitochondrial apoptotic pathway [185]. On the other hand, cytosolic Ca^2+^ levels are elevated in KD-mimicking oligodendrocytes, inducing transient mitochondrial membrane hyperpolarization, followed by depolarization and apoptosis [188].

Impaired lysosomal Ca^2+^ homeostasis is observed in NPC1-inactivated cells as a result of Sph accumulation. Sph accumulation is the first event occurring after NPC1 inactivation, followed by an alteration in lysosomal Ca^2+^ levels caused by Sph storage [126]. This Ca^2+^ defect is caused by altered nicotinic acid adenine dinucleotide phosphate (NAADP)-mediated lysosomal Ca^2+^ signaling. NAADP is a strong Ca^2+^-releasing second messenger that targets lysosomal Ca^2+^ channels to modulate Ca^2+^ levels required for proper endolysosomal trafficking [189]. Therefore, a defect in this pathway eventually leads to altered endocytosis and vesicular fusion in NPC1 fibroblasts, macrophages, astroglia, and cerebellar Purkinje cells [126]. Under normal circumstances, Ca^2+^ is released from the lysosomal lumen into the cytosol via TRPML1. Its release stimulates the kinase activity of mTORC1 complex [190], and the Ca^2+^-dependent phosphatase calcineurin [191]. Activated mTORC1 phosphorylates multiple targets including the transcription factor TFEEB that becomes inactive upon phosphorylation [192]. Calcineurin, on the other hand, dephosphorylates and translocates TFEEB to the nucleus, which allows the transcription of target genes responsible for autophagy regulation, and lysosomal biogenesis and function [191]. Although TRPML1-mediated Ca^2+^ release may appear to simultaneously activate and inhibit the activity of TFEEB, it may be an important factor in the fine-tuning of TFEEB activity and nuclear translocation. The accumulation of lysosomal SM in NPC cells inhibits the activity of TRPML1 [98], causing inhibition of lysosomal trafficking [193]. As a result, drugs regulating the expression of TFEEB or other lysosomal-trafficking regulators may be a potential future therapeutic strategy for LSDs [6].

Taken together, these findings suggest that altered Ca^2+^ homeostasis in the ER, mitochondria, or lysosomes is involved in LSD pathogenesis due to signaling crosstalk and physical contact among the three organelles (Fig. 4).

#### Impaired Autophagy

Dysfunctional autophagy is a principal pathophysiological mechanism in multiple LSDs [194]. Lysosomal autophagy is the process by which the cell degrades its macromolecules and damaged/ injured organelles to maintain physiological and cellular homeostasis. There are four levels of the autophagic degradative pathway: autophagosome formation, autophagosome-lysosome fusion, autophagosome degradation, and lysosomal membrane recycling [194]. A defect in one or more leads to autophagic impairment. In several sphingolipidoses, impaired autophagosome degradation leads to increased levels of the autophagosome marker LC3-II, damaged mitochondria, and polyubiquitinated proteins, which are putative stimulators of apoptotic cell death [195]. In addition to the accumulated autophagosome levels, Beclin-1, a major autophagy regulator, increases [196]. This suggests that the cell attempts to compensate for impaired autophagic degradation by creating more autophagosomes, which in turn increases the amount of damaged material inside the cell, further worsening lysosomal trafficking [197].

As a consequence of impaired autophagy and autophagosome accumulation, cellular levels of the autophagic p62/ sequestosome-1 increase [198]. p62/ sequestosome-1 is a receptor that recognizes ubiquitinated proteins and selectively targets them for autophagy [199]. In the brains and astrocytes of GD mouse models, there is an increase in p62 along with dysfunctional mitochondria, ubiquitinated proteins and insoluble ɑ-synuclein, indicative of aberrant autophagy [200, 201]. Moreover, a *Drosophila* neuronopathic GD model shows severe lysosomal defects, neurodegeneration and reduced lifespan [202]. In composite, these results suggest that dysfunctional autophagic flux is a central mechanism underlying neurodegeneration in several LSDs.

#### Lysosomal Membrane Permeabilization and Cell Death

Lysosomal membrane permeabilization (LMP) causes lysosomal contents to be extruded into the cytosol, eventually leading to cell death [203]. LMP is activated by multiple factors, including oxidative stress and cytosolic Ca^2+^ [204, 205]. The lysosomal membrane is more susceptible to oxidative attack than other membranes: lysosomes contain high levels of iron because they are the degradation site for heme. Intra-lysosomal iron reacts with hydrogen peroxide to produce free radicals that destabilize the lysosomal membrane, leading to its permeabilization [204]. In the lysosomal membrane, Hsp70 binds to BMP and inhibits LMP. When cytosolic Ca^2+^ levels increase, the mammalian cysteine protease Calpain is activated. Calpain then cleaves Hsp70, and thus sensitizes lysosomal membranes to LMP. This eventually leads to neuronal cell death in a cathepsin-dependent manner [205]. LMP can eventually result in lysosomal damage, autophagosome accumulation, and ultimately cell death [206]. Interestingly, Cer channels are present in lysosomal membranes, which may also lead to permeabilization [207], as they do in MOM [208, 209].

As cathepsins exit the lysosomes, cell death ensues. Although liberated cathepsins function optimally at acidic pH, some cathepsins (*e.g.*, B, D, and L) can perform their proteolytic cleavage at neutral pH. These cathepsins can proteolytically activate specific molecules that are involved in cell death cascades, including BH3 and Bid, activate other members of the Bcl-2 family (*e.g.,* Bak, and Bax). Activated Bak and Bax can, in turn, activate mitochondrial permeabilization and cytochrome *c* release, thereby initiating mitochondrial caspase-mediated cell death [210]. Other BH3 proteins such as Noxa are also involved in LMP-activated cell death [211]. Some caspase-independent cell death pathways, such as the RIPK1 and RIPK3-dependent pathways, can be activated by LMP as well [212].

LMP-induced cell death is observed in models of several sphingolipidoses. LMP induces neurodegeneration in aSMase-knockout mice (NPD-A mouse models) through the release of cathepsin B, which causes autophagic impairment and cell death [206]. Similarly, microglia and astrocytes of neuronopathic GD mouse models show translocation of cathepsin D to the cytosol [213]. RIPK3-deficient mice are protected against chemically-induced neuronopathic GD by the irreversible inhibitor of GCase, conduritol B epoxide [214]. This suggests that LMP participates in the RIPK3-mediated cell death in neuronopathic GD. Moreover, an absence of caspase activity in combination with elevated levels of RIPK1 and RIPK3 in neural cells of *GBA*-deficient mice suggests that the mode of neuronal cell death is independent of caspases, even at times of advanced neurodegeneration. The elevated levels of RIPK1 in microglial cells also suggests its participation in neuroinflammation [214]. However, these caspase-independent cell death pathways are not observed in GM1 gangliosidosis, NPC, and Sandhoff disease models [214]. Because lysosomal destabilization contributes to sphingolipidosis pathology, LMP inhibition by the chaperone Hsp70 could be a potential therapeutic strategy, warranting further investigation in current clinical trials [203].

#### ER Stress and the Unfolded Protein Response

A quality control system in the ER is responsible for determining whether a certain protein is properly folded. If a protein fails to adopt a proper conformation, it accumulates, creating ER stress. To achieve homeostasis, cells activate the UPR. The UPR can be activated via three ER transmembrane proteins that represent the major sensors in eukaryotes: IRE1, ATF6, and PERK. ER chaperones are key players in the UPR. They bind to unfolded proteins and/ or translocate them to the cytoplasm. If the UPR fails to achieve homeostasis by decreasing ER stress, it eventually leads to apoptosis. The UPR can also be activated indirectly by depleted ER Ca^2+^ levels [215].

The activation of the UPR was documented in GM1 gangliosidosis mouse models, in which the upregulation of the transcriptional regulator CHOP and the chaperone BiP were observed. GM1 accumulation in the models leads to decreased Ca^2+^ levels in the ER through SERCA inhibition. This may activate the UPR, leading eventually to apoptotic cell death [216]. Furthermore, patients of other sphingolipidoses including GD-type 2 and Tay-Sachs disease have increased UPR [217].

The UPR participates in the pathology of KD in a mutation-dependent manner. Different mutations of the *GALC* gene stimulate varying combinations of UPR sensors, resulting in varying residual activities of the mutated enzyme, and leading to differential pathological severity [215]. These results suggest that increased translocation of the enzyme to the cytosol decreases its trafficking to the lysosome, and thus decreases its residual activity, eventually leading to more sever pathological manifestations.

#### Impaired Lipid Trafficking and Endocytosis

Endocytosis and vesicular trafficking rely largely on SL and Chol levels. Lipid mis-sorting is a common feature of sphingolipidoses. For example, caveolae-internalized BODIPY-labeled LacCer accumulates in endosomes/ lysosomes of LSD fibroblasts due to faulty intracellular Chol distribution [218]. Similar mis-sorting of BODIPY-LacCer is observed in GCase-inhibited cells, resulting in increased storage of GlcCer [219]. Such mis-sorting was reversed by lowering Chol and GlcCer levels in LSD fibroblasts and GD cell models, respectively [13], suggesting that impaired lipid trafficking is a secondary consequence of Chol accumulation in multiple LSDs. Impaired trafficking is not exclusive to membrane lipids, as it is also observed for membrane proteins [152]. Trafficking of both mannose 6-phosphate and transferrin receptors is impaired in MLD mouse models [220]. In NPC mouse models, mannose 6-phosphate receptors are concentrated in late endosomes, suggesting that there are more endosomal pools of plasma membrane receptors in multiple sphingolipidoses [221].

There is a strong correlation between increased lipid storage and impaired endocytosis. In models of four sphingolipidoses (NPD-A, NPC, Fabry disease, and GD), endocytosis is disrupted in a Chol-dependent manner. The activities of pinocytosis, macropinocytosis, clathrin-dependent, and caveolin-dependent-endocytosis, as well as intracellular lipid and protein trafficking are affected [222]. Hence, targeting pathways involved in lipid and protein trafficking could serve as potential therapeutic approaches to alleviate pathogenesis in sphingolipidoses [223].

#### Mitochondrial Function and Oxidative Stress

The cellular physiological integrity of non-mitotic neural cells is dependent on the coordination between the degradative role of lysosomes and the energy-production capacity of mitochondria. Therefore, any lysosomal impairment could affect mitochondrial morphology, trafficking, and/ or degradation, particularly in neural cells [162, 224]. Mitochondrial morphological abnormalities are accompanied by dysfunctional mitochondrial respiration and a reduction in mitochondrial membrane potential in neurons and astrocytes of neuronopathic GD mouse models [225]. Neurons of NPC1 mouse models have smaller mitochondria with decreased membrane potential and ATP production. Human embryonic stem-cell derived neurons with decreased NPC1 activity have fragmented mitochondria and decreased activities of mitochondrial proteins, but no change in membrane potential [226].

Trafficking of mitochondria towards energy-requiring regions of the cells is an important aspect of mitochondrial function, especially in neurons that have long axons [162]. Such trafficking requires the spatiotemporal fine-tuning of intracellular Ca^2+^ levels [227]. Because Ca^2+^ ion homeostasis is impaired in several LSDs, mitochondrial trafficking is also expected to be impaired. Indeed, psychosine-treated neurons show a reduced rate of mitochondrial movement in axons *in vitro*, suggesting a potential pathogenic mechanism of KD [150].

Mitophagy is a specialized autophagic pathway that removes abnormally-shaped and fragmented mitochondria [162]. Mitophagy begins with the accumulation PINK1 on the mitochondrial outer membrane (MOM), triggered by the reduction of mitochondrial membrane potential. PINK1 undergoes autophosphorylation followed by phosphorylation and recruitment of Parkin, which ubiquitinates MOM proteins. Ubiquitinated proteins, in turn, recruit both Nuclear domain 10 protein 52 and optineurin, which bind to 1A/1B light chain 3 (LC3), a microtubule-associated protein that triggers autophagosome formation around dysfunctional mitochondria. Meanwhile, Parkin also interacts with Beclin-1, further triggering mitophagy [228, 229]. Therefore, when mitophagy is aberrant, dysfunctional mitochondria accumulate.

Dysfunctional mitochondria have disrupted respiratory chains that accumulate ROS, causing oxidative damage to cellular DNA, lipids, and proteins, events that characterize several LSDs [226]. In B lymphocytes extracted from human NPD-B cells, there are significant changes in autophagosome accumulation and mitochondrial fragmentation, along with induction of mitophagy and aberrant lipid trafficking [230]. GD fibroblasts and activated GM1/ GM2 gangliosidoses microglia/ macrophages have upregulated apurinic endonuclease 1, which is an oxidative damage DNA repair enzyme [231], and elevated inducible nitric oxide synthase and nitrotyrosine levels [232]. Also, NPC fibroblasts contain oxidized lipids, proteins, and DNA [233]. Similarly, the brains of GM2 gangliosidosis mice suffer oxidative damage and the induction of cell death [234]. Permeabilization of MOM via Cer channels [3, 208, 235–237] that are exacerbated by pro-apoptotic Bcl-2 family proteins [238] is another mechanism relating mitochondrial health to LSDs. SL pathology can therefore impact mitochondrial apoptosis. Thus, disrupted mitochondrial clearance and oxidative stress appear to be common pathological pathways in several LSDs. Systematic investigation of the involvement these pathways is needed to be able to target them using novel therapeutic approaches.

## Therapeutic Approaches to Sphingolipidoses

**Fig. 5:**
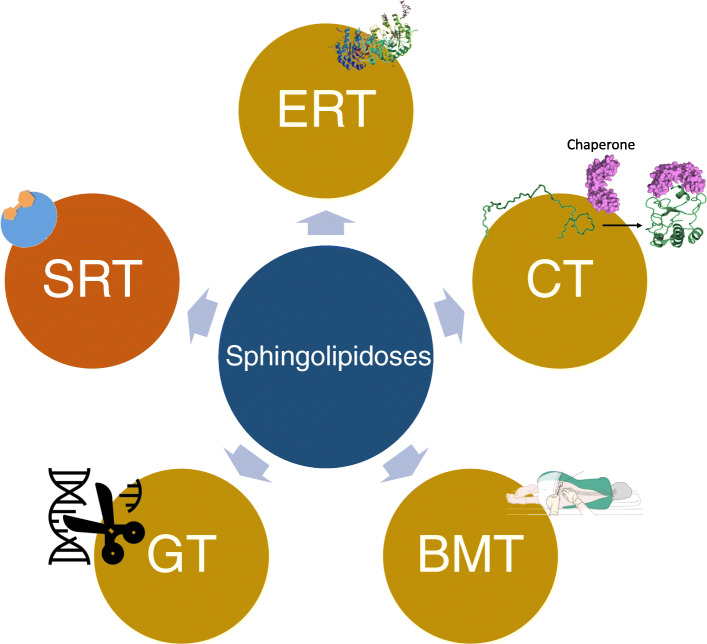
General therapeutic strategies for the treatment of sphingolipidoses. There are five main therapeutic approaches to treat sphingolipidoses. Substrate reduction therapy (SRT, orange) involves the prevention of influx of substrates into the lysosome, to lower synthesis of the accumulating substance. The other treatment strategies (gold) involve enhancing the activity of the missing or malfunctioning enzyme. Enzyme replacement therapy (ERT) uses purified enzyme to reverse the pathology (the crystal structure of α-GAL is shown). Chaperone therapy (CT) is used to assist the folding of misfolded enzymes to be targeted to the lysosome. Bone marrow transplantation and stem cell transplantation (BMT) are used to supply the body with the correct form of the missing enzyme, and gene therapy (GT) is used to modify the genes responsible for the aberrant phenotype

Treatments for SL-related LSDs are based on two major concepts. Either the treatment is targeted to decrease the concentration of accumulating substrates (depicted in Fig. 5, orange circles) or to reduce the rate of their synthesis (Fig. 5, gold circles). The former strategy focuses on increasing the residual activity of the hydrolytic enzyme by increasing the concentration of wild-type enzymes above-threshold levels. This can be achieved via multiple approaches [239]. The latter strategy aims to reduce the influx of the substrate to the lysosome.

### Enzyme Replacement Therapy (ERT)

ERT supplies the active enzyme exogenously to patients weekly or biweekly [240]. Deficient cells take up the recombinant enzyme by receptor-mediated endocytosis, and then transport it to lysosomes where it will function. Therapeutic enzymes are derived from genetically-modified plants, model animals, or human cells [241]. The mannose 6-phosphate receptor system, found in nearly all cells, is generally used to target the enzyme for uptake.

ERT was found to improve the course of many LSDs by reducing the accumulating substance, decreasing organomegaly, and ameliorating the function of many organs [241]. For example, most GD type 1 patients respond well to ERT using several recombinant enzymes such as imiglucerase. ERT patients show improved platelet counts and hemoglobin concentrations, decreased splenomegaly, skeletal pain, and bone-related symptoms within six months of enzyme administration. Other disease manifestations, however, need longer periods to improve. Notably, because the recombinant enzymes cannot cross the BBB, they do not improve the neurological manifestations of types 2 and 3 GD [242]. Nevertheless, a recent study in a neuronopathic GD mouse model used a non-invasive, CNS-selective delivery system mediated by nanovesicles of SapC and dioleoylphosphatidylserine to deliver GCase to deficient cells of the CNS. Treated mice showed improvements in neurodegeneration, brain inflammation, and associated phenotype compared to controls [243].

ERT was also tested on Fabry disease patients and MLD mouse models. For MLD, intravenous administration of Metazym (recombinant human ASA) did not show a beneficial effect on the CNS- and PNS-related manifestations [244]. However, in humanized ASA knockout MLD mouse models, a three-fold decrease in PNS and CNS sulfatide accumulation was achieved by increasing the catalytic rate constant of the intravenously-administered enzyme [245]. Also, recombinant human ASA was used in clinical trials and is under development for intrathecal administration in patients with late-infantile and juvenile MLD [246]. The intrathecal injection was also shown to be a potential approach for treating infantile NPC [46]. For Fabry disease, on the other hand, two α-GAL preparations (agalsidase-α and agalsidase-β) were authorized by the European Medicines Agency to treat the disease, as they were found to help in Gb3 clearance, pain improvement, and decreased occurrence of complications upon prolonged treatments [247, 248]. Nonetheless, both enzymes have limited effects on cerebral, renal, and cardiac disease manifestations [249–251].

ERT was shown to decrease visceral but not neurological manifestations of Farber’s disease in mouse models even though to date, no cure for the disease is available [252]. The variability in the clinical efficacy of ERT can be attributed to the wide variation of pathological manifestations exhibited by patients, and the immune response of patients toward the recombinant enzyme, which may limit the efficacy of the treatment [241].

### Enzyme Enhancement Therapy/ Chaperone Therapy

Newly-synthesized enzymes must adopt the correct conformation to function properly. Otherwise, misfolded enzymes are degraded in the proteasome. Abnormal folding may result from genetic mutations that characterize multiple LSDs, eventually preventing the enzyme from reaching its destination and performing its function. Some missense mutations, however, may produce mutant enzymes whose function may be restored at least partially by the use of small stabilizing molecules, or chaperones [241]. The efficacy of this “chaperone therapy” (CT) was first investigated *in vitro* using different mutant forms of α-GAL, the deficient enzyme in Fabry disease. 1-deoxygalactonojirimycin (migalastat) is an analog of the natural substrate [253], and binds reversibly to the active site of the enzyme with a very high affinity, stabilizing it and resulting in decreased levels of the storage material, Gb3 [254]. *In vivo* studies also showed decreased Gb3 levels in α-GAL knockout mouse models upon oral administration of migalastat [255].

Multiple carbohydrate analogs and non-carbohydrate molecules that increase the activity of the defective GCase in GD cells have been evaluated [256]. Isofagomine (afegostat tartrate) and ambroxol, were found to be promising in preclinical and early clinical studies, respectively [257]. Isofagomine binds mutant (and wild-type) GCase stabilizing it, thus leading to the increased catalytic activity of the enzyme in the bone, spleen, liver, and lung of non-neuronopathic GD transgenic mouse models [258]. Upon oral administration, isofagomine decreased neurological manifestations and neuroinflammation in neuronopathic GD mouse models [259]. Ambroxol, on the other hand, was shown stablilize wild-type GCase under high-temperature conditions. Its affinity to GCase increased at lysosomal pH levels *in vitro* [260]. *In vivo*, subcutaneous injections of ambroxol increased GCase levels in the spleen and liver of transgenic non-neuronopathic GD mice [261]. In another sphingolipidosis, β-gal activity was enhanced in transgenic animal models of GM1 gangliosidosis by the small molecules 5N,6S-(N'-butyliminomethylidene)-6-thio-1-deoxygalactonojirimycin and *N*-octyl-4-epi-β-valienamine [262].

Despite the promising efficacy of CT in treating multiple LSDs, its use faces challenges that need evaluation in future research. These include insufficient increases in enzymatic activity that result in non-significant benefits, and the mutation-specific unresponsiveness of some defective enzymes to molecular chaperones [263].

### Bone Marrow and Stem Cell Transplantation

Cell-mediated therapy is based on using stem cells as delivery vehicles to carry either normally -expressed or genetically-overexpressed enzymes that are deficient in host cells. These cells can self-renew and differentiate into healthy tissue, to produce the deficient enzyme, restoring lysosomal function and preventing the accumulation of storage material [264].

Hematopoietic stem cell (HSC) transplantation/ bone marrow transplantation (BMT) was the first and only method to treat LSDs before the development of ERT. HSCs are multipotent progenitor cells that can differentiate into all types of blood cells. Compensation for the defective enzyme is achieved in the neurons of the CNS and PNS via the partial replacement of the host’s microglial cells by donor HSCs. Donor HSCs are derived from peripheral blood, bone marrow, or umbilical cord blood, and can cross the and blood-nerve barrier and differentiate into fully functional microglia/ macrophages [265]. For instance, umbilical cord blood of unrelated donors can be used to treat infants with infantile KD. Such an approach showed increased blood GALC levels, progressive myelination of CNS neurons, age-appropriate cognitive function, and developmental skills before the expression of pathological symptoms in newborns. However, patients showed mild-to-severe and mild-to-moderate delays in motor function and expressive language, respectively [266].

In minimally symptomatic Farber disease patients, on the other hand, BMT improved peripheral manifestations but failed to improve neurological degradation [267]. Nevertheless, stably engrafted allogenic HSCs showed a slow substitution of ASA deficient cells, leading to belated disease stabilization by 12-24 months. Therefore bone-marrow-derived HSCs is inappropriate for treating patients with late infantile MLD [268]. This may be attributed to the long lifespan of microglia, which slows their repopulation in the brain [152].

An alternative strategy is the use of multipotent neural stem cells (NSCs) directly delivered to the brain by intracranial injection. *Ex vivo* genetic modification is used to increase the expression of the required enzyme before transplantation [264]. This approach has been evaluated in Tay-Sachs mouse models and showed increased levels of β-hexosaminidase in their brains [269]. Delayed onset of disease and reduced storage were also shown in neonatal Sandhoff mouse models treated with unmodified murine NSCs [270]. Moreover, unmodified human and immortalized murine NSCs also showed a therapeutic effect when used on neonatal NPD-A mice, resulting in decreased brain Chol levels and neural and glial vacuolation [271]. Neonatal NPC1 diseased mice showed delayed ataxia onset and increased Purkinje cell survival upon treatment with NSCs [272]. Other types of stem cells like mesenchymal stem cells (MSCs) can also be used to treat NPC1, as bone marrow-derived MSCs were shown to decrease inflammation and apoptosis in the brains of NPC1 diseased mice [273].

Although primary research findings show promising therapeutic results of stem cell therapy, multiple factors should be optimized before applying it to human brains. These include but are not limited to determining the dose of cells and target sites of injection; evaluating proper *ex vivo* genetic modification of cells to maximize the amount of cross-corrected enzyme; characterizing a nontumorigenic human stem cell source, and immunosuppressing the patient for allogenic transplants [264]. The latter issue could be optimized by using modified human induced pluripotent stem cells (iPSCs) produced by introducing embryogenesis-related genes to adult somatic cells, as they are derived from the patient’s fibroblasts. However, iPSC reprogramming has its challenges that need to be overcome first [274].

### Gene Therapy

Because most sphingolipidoses are single-gene disorders, with no extremely complex regulatory mechanisms, gene therapy can be considered as a potential therapeutic approach for such diseases. Gene therapy involves two general approaches, *in vivo* and *ex vivo* [275]. *Ex vivo* gene therapy involves genetically modifying stem cells before transplantation. Genetic modification is required either to modify the gene of the mutant enzyme in patient-derived stem cells to avoid the potential patient immune response, or to overexpress the enzyme in the transplanted stem cells [241]. Lentiviral vectors were used to transfer the *ASA* gene into HSCs derived from three children affected by MLD. Treated children did not show any pathological manifestations, even after the period of predicted onset [276]. *In vivo* gene therapy directly delivers the gene into a specific organ using a vector. The enzyme resulting from the transferred gene can be produced and secreted to be taken up by other cells via the mannose 6-phosphate receptor. Because the enzyme cannot cross the BBB, this approach was mostly studied in peripheral organs. Whereas some studies using direct injections of adeno-associated virus vector (AAV9) into the CNS were shown to be effective and safe [275], other studies proved otherwise. Direct injection of AAV2-human aSMase into the CNS of non-human primates demonstrated dose-dependent toxicity. High dosage of the viral vector induced significant motor deficits in primates. Moreover, aSMase delivered by AAV2 lacked intercellular transport from transfected cells to other cells, which would affect its therapeutic benefit [277].

Interestingly, *GBA* gene was systematically delivered to neuronopathic murine GD models via intraperitoneal injection of AAV9 at postnatal day 5. Treated models showed improved GCase activity, increased lifespan, and improved neurological symptoms [278]. In addition, multiple administration methods were used to transfer *NPC1*-gene-containing-AAV9 to NPC1-deficient mice. Intra-cardiac injection at postnatal day 24 was found to extend the lifespan by 32% [279]. Intracerebroventricular injection directly after birth also resulted in improvement in liver pathology and increased lifespan by 111% [280].

Recently, gene editing using CRISPR/ Cas9 has been employed to create models of sphingolipidoses, and in some cases, for therapeutic use. In β-gal-deficient iPSCs, the aberrant gene was edited by targeting *GLB1* exons 2 and 6. Treated iPSCs showed increased β-gal activity and reduced GM1 ganglioside storage, demonstrating the predicted efficacy of the gene therapy-based treatment in GM1 gangliosidosis [102]. Moreover, the activity of α-GAL in the fibroblasts of Fabry disease patients was restored by CRISPR/ Cas9 therapy. Single guide RNAs were used to delete the GLA IVS4 + 919 G➔A mutation responsible for disruption of normal RNA splicing, resulting in an enzyme with no catalytic activity. Upon editing, fibroblasts showed increased α-GAL activity and decreased Gb3 storage levels [281]. CRISPR/ Cas9 was also used to gene-correct fibroblast-derived iPSCs prepared from infantile Sandhoff patients. To investigate the efficacy, a cerebral organoid was formed from edited and non-edited iPSCs to mimic neurodevelopment in the first trimester. GM2 accumulation and high cellular size were only detected in non-edited Sandhoff iPSCs [282]. Finally, a recent study showed that engineering human neural stem cells using CRISPR/ Cas9 can be used to cross-correct fibroblasts of KD patients *in vitro*. Transplantation of such cells in oligodendrocyte-mutant, shiverer-immunodeficient mice resulted in neural stem cell differentiation, with an overexpressed-GALC phenotype [283].

### Substrate Reduction Therapy

In contrast to the earlier therapies that focus on the increase in enzymatic activity, SRT is based on reducing the influx of the accumulating substrate to the lysosome by reducing its biosynthetic rate [284]. The first proof-of-principle genetic model of this approach was a mouse model created by crossbreeding a Sandhoff diseased mouse with another having a defective GM2/GD2-synthase. The resulting offspring showed much longer lifespans, although the model suffered from accumulation of other oligosaccharides that resulted in late-onset neurological manifestations [285]. Using small inhibitor molecules of SL biosynthetic enzymes reduces substrate influx into lysosomes. An example is the use of *N*-butyldeoxynojirimycin (miglustat) as a modest inhibitor of GlcCer synthase that produces GlcCer, a common precursor to many GSLs accumulating in multiple sphingolipidoses. Its efficacy has been tested in Tay-Sachs mice. Moreover, it is currently being used as a potential drug treatment for patients with non-neuronopathic GD [286]. Even though it was initially thought to be an effective inhibitor of the enzyme to reduce substrate incorporation into the lysosome, recent evidence showed that it may be working as a chaperone for GCase [287, 288]. For chronic neuronopathic GD patients, combination therapy of intravenous ERT and oral miglustat was shown to prevent neurological symptoms [289]. Combination therapy using miglustat and NSAIDs to further reduce neuroinflammation was shown to increase the lifespan of miglustat-treated Sandhoff model mice [178]. In addition to miglustat, another FDA-approved GlcCer synthase partial inhibitor, eliglustat tartrate, was shown in its Phase 2 trial to decrease mean volumes of spleen and liver, and increase platelet count and hemoglobin concentration in patients of GD type 1, with about 98% of adverse effects being mild or moderate effects [290]. Moreover, Genz-682452, a novel GlcCer synthase inhibitor with CNS access, was previously shown to be a potential Fabry disease combinatorial treatment with ERT. It was investigated to treat brain manifestations of GD type 3, and was shown to decrease the severity of gliosis and the storage of brain glycolipids by 20% in two neuronopathic GD type 3 mouse models [291].

GlcCer synthase inhibitors can be used to treat GSL-based sphingolipidoses, but not other diseases like NPD-A, NPD-B, MLD, and KD [292]. In NPC mice, however, miglustat helped to prolong lifespan and decrease GSL accumulation in the brain, and was approved in Europe as a treatment for neurological manifestations of juvenile and adult GD and NPC [293]. Notably, hydroxypropyl-beta-cyclodextrin is a substrate reduction drug for NPC currently in phase 3 clinical trial, and offers hope for the cure of the disease [294–296]. This drug also inhibited cerebellar Purkinje cell damage in NPC disease mouse models [297], thus alleviating disease symptoms. Despite lack of an inhibitor of CGT, inhibition of 3-ketosphinganine synthase by L-cycloserine was shown to increase the lifespan, and to decrease astrocyte gliosis and macrophage infiltration in KD mouse models [292].

Taken together, these findings suggest that using SRT has advantages over ERT, because the small molecule inhibitors used are orally administered, easier to produce than recombinant enzymes, can cross the BBB easily to treat neurological pathology, and cost less.

## Conclusions

A rapid search of articles on sphingolipidoses shows a remarkable and exponential rise in publications since the 1940s. Currently, more than 15,000 articles address the topic. At present, significant progress has been made in the understanding the underlying molecular mechanisms governing the pathogenesis of sphingolipidoses. Therapeutically, myriad options are available to combat these debilitating diseases and increasingly more patients are benefiting from them. Combinatorial therapeutic options are currently being used for better efficacy, improving symptoms and extending quality of life. Novel use of CRISPR/ Cas9 in gene editing and gene therapy offers hope for future disease eradication.

We believe we have presented a thorough picture of a subset of lysosomal storage diseases that involve aberrant SL metabolism, and possible treatment avenues of these diseases. SL research is thriving, and the contribution from scientists worldwide is making enormous leaps in the understanding of both basic SL biochemistry and applications in health and disease.

## Data Availability

Not applicable

## Abbreviations

α-GAL: α-Galactosidase A
β-gal: GM1-β-galactosidase
β-hexoseaminidase: β-*N*-acetyl-hexoseaminidase
AAV9: Adeno-associated virus vector
ASA: Arylsulfatase A
aSMase: Acid sphingomyelinase
BBB: Blood-brain barrier
BMP: Bis(monoacylglycero)phosphate
BMT: Bone marrow transplantation
CGT: Ceramide galactosyltransferase
CNS: Central nervous system
CT: Chaperone therapy
Cer: Ceramide
Chol: Cholesterol
ER: Endoplasmic reticulum
ERT: Enzyme replacement therapy
GCase: Glucosylceramide-β-glucosidase
GD: Gaucher disease
GM2A: GM2-activator protein
GSL: Glycosphingolipid
GalCer: Galactosylceramide
GALC: Galactosylceramide-β-galactosidase
Gb3: Globotriaosylceramide
GlcCer: Glucosylceramide
GlcSph: Glucosylsphingosine
HSCs: Hematopoietic stem cells
IL: Interleukin
iPSCs: Induced pluripotent stem cells
KD: Krabbe disease
LMP: Lysosomal membrane permeabilization
LSDs: Lysosomal storage diseases
LacCer: Lactosylceramide
Lyso-Gb3: Globotriaosylsphingosine
MEFs: Mouse embryonic fibroblasts
MLD: Metachromatic leukodystrophy
MOM: Mitochondrial outer membrane
MSCs: Mesenchymal stem cells
NAADP: Nicotinic acid adenine dinucleotide phosphate
NPC: Niemann Pick disease type C
NPD-A, NPD-B: Niemann Pick disease types A, B
NSAIDs: Non-steroidal anti-inflammatory drugs
NSCs: Neural stem cells
PKC: Protein kinase C
PMCA: Plasma membrane Ca^2+^-ATPase
PNS: Peripheral nervous system
psychosine: Galactosylsphingosine
ROS: Reactive oxygen species
Sph: Sphingosine
S1P: Sphingosine 1-phosphate
SERCA: Sarco/ endoplasmic reticulum Ca^2+^-ATPase
SL: Sphingolipid
SM: Sphingomyelin
SMase: Sphingomyelinase
Saps: Saposins
SRT: Substrate reduction therapy
TLR: Toll-like receptor
TNF-α: Tumor necrosis factor α
UPR: Unfolded protein response
